# Efficacy and safety of essential-oil-containing mouthrinses for plaque and gingivitis control in people with diabetes: an examiner-blind, randomised controlled trial

**DOI:** 10.1038/s41405-026-00420-5

**Published:** 2026-06-22

**Authors:** Kimberly Milleman, Mary Lynn Bosma, James A. McGuire, Ruth Chen, Alicia DelSasso, Patricia Gorecki, Abbie Yoder, Jeffery Milleman

**Affiliations:** 1 Clinical Operations, Salus Research Inc., Fort Wayne, IN USA; 2 Oral Healthcare, Kenvue Brands LLC, Summit, NJ USA

**Keywords:** Gingivitis, Plaque, Oral hygiene, Oral-health-related quality of life, Periodontitis

## Abstract

**Aims:**

To evaluate the anti-plaque and anti-gingivitis efficacy and the safety of essential-oil-containing mouthrinses in people with diabetes, a group at high periodontal disease risk.

**Materials and methods:**

This Phase IV, examiner-blind trial randomised 154 adults with type 1 (*n* = 15) or type 2 (*n* = 139) diabetes to use alcohol-containing essential oil (ACEO), alcohol-free essential oil (AFEO), or negative control mouthrinses alongside twice-daily brushing for 12 weeks. Assessments included a plaque index (TPI), gingival index (MGI), bleeding index (EBI), participant survey and adverse event monitoring.

**Results:**

The essential-oil-containing mouthrinses (ACEO/AFEO) significantly reduced the primary endpoints of mean TPI (24.7%/13.2%) and mean MGI (37.2%/32.8%), versus the negative control, at Week 12 (*p* < 0.001). Both mouthrinses reduced EBI (68.7%/72.6%; *p* < 0.001), and participants reported better oral health experiences, versus negative control, at Week 12. Overall, 26.2% of participants were unaware of their elevated oral health risk due to diabetes, and 37.9% reported receiving no oral health guidance from healthcare providers. The mouthrinses showed good soft tissue tolerance.

**Conclusions:**

Essential-oil-containing mouthrinses significantly improved periodontal health in people with diabetes, when compared with a negative control rinse, and may serve as a valuable adjunct to mechanical oral hygiene in this population. The identified knowledge gap highlights the need for collaboration between primary, diabetes and dental practitioners to enhance patient outcomes through integrated care.

## Introduction

People with diabetes have a substantially higher prevalence of periodontal disease than people without diabetes (58.0% versus 37.6%, respectively, in the USA), making oral health an important concern in diabetes management [1]. Diabetes mellitus and periodontal disease have a bidirectional relationship: diabetes increases the risk of periodontal disease, while periodontal disease adversely affects glycaemic control and exacerbates diabetes-related complications [2, 3].

Gingivitis, the earliest form of periodontal disease, is characterised by inflammation of the marginal gingival tissue surrounding teeth, with gingival bleeding being a common symptom. While gingivitis affects most adults in the general population, with increased prevalence in people with diabetes, it is fully reversible on improvement of oral hygiene [4–6]. The accumulation of bacteria and debris between the gum line and tooth, known as dental plaque, is the primary aetiologic factor for the development of gingivitis [5].

Daily mechanical oral hygiene, such as toothbrushing and interdental cleaning, is fundamental for the effective removal of dental plaque and the prevention of both gingivitis and progression to periodontitis – a chronic, destructive, irreversible inflammatory disease state that severely impacts individuals and health services [5, 7–9]. In certain patient groups who may have challenges in achieving optimal plaque control, such as those with diabetes, adjunctive chemical plaque control agents, such as antimicrobial mouthrinses, may provide additional benefits [7].

Essential-oil-containing mouthrinses (with and without alcohol) are recognised as being clinically effective against plaque, gingivitis and gingival bleeding, when used alongside mechanical oral hygiene, in the general population [10–12]. These mouthrinses typically contain a combination of essential oils, such as eucalyptol, menthol, methyl salicylate and thymol [13], which exhibit multifaceted antimicrobial properties, including disruption of bacterial cell walls and biofilms, inhibition of enzymatic activity and suppression of bacterial proliferation [14].

Mouthrinses containing the antimicrobial agents cetylpyridinium chloride (CPC) and chlorhexidine (CHX) also have well-established efficacy, when used in conjunction with mechanical methods, for controlling plaque and gingivitis in the general population [15–19]. All these antimicrobial rinses exhibit good safety profiles in the general population; however, CPC and CHX can cause brown staining of the teeth, tongue and/or restorations, and CHX can cause supragingival calculus formation and a change in taste sensation [13]. These side effects could potentially impact patient adherence [20, 21], highlighting the importance of considering patient experiences with mouthrinses in clinical practice. In the USA, essential-oil- and CPC-containing mouthrinses are widely available over the counter, while those containing CHX are only available on prescription [13].

There is a paucity of data on essential-oil-containing mouthrinses in people with diabetes. The objective of the current study is to compare the efficacy and safety of twice-daily rinsing with an essential-oil-containing mouthrinse (with or without alcohol) with a negative control rinse, both used in conjunction with mechanical oral hygiene, for the control of plaque, gingivitis and gingival bleeding in people with type 1 or type 2 diabetes mellitus and mild-to-moderate gingivitis and plaque. The study also included a survey to capture participants’ experiences of the study mouthrinses and gain insights into their oral health awareness.

## Materials and methods

### Trial design

This was a Phase IV, 12-week, single-centre, examiner-blind, randomised, parallel-group, controlled clinical trial (NCT04449952) conducted from December 2019 to March 2020 at Salus Research Inc. (Fort Wayne, IN, USA), an American Dental Association–qualified site [22]. The study was conducted in accordance with the Declaration of Helsinki and Good Clinical Practice guidelines, and all participants provided written informed consent. The trial protocol was approved by the IntegReview Institutional Review Board, Austin, TX, USA (25 November 2019; amended 16 December 2019; see Supplementary appendix for more details).

### Participants

The trial enroled participants ≥18 years of age with investigator-verified type 1 or type 2 diabetes mellitus (see protocol amendment in the Supplementary Information). Key inclusion criteria were good general and oral health; adequate oral hygiene and a minimum of 20 gradable teeth; glycated haemoglobin (HbA1c) of <7.0% for type 1 diabetes or <8.0% for type 2 diabetes; and scores of ≥1.95 on the Turesky modification of the Quigley–Hein Plaque Index (TPI) [23], ≥1.85 on the Modified Gingival Index (MGI) [24], and ≥10% of sites that bled on the Expanded Bleeding Index (EBI) [25]. Key exclusion criteria were dental prophylaxis or use of chemotherapeutic oral care products within 4 weeks prior to baseline, and fixed or removable orthodontic appliances or removable partial dentures (see Supplementary Information for full eligibility criteria).

### Interventions

At baseline, after abstaining from oral hygiene for between 8 and 18 h, screened participants received an oral examination that included TPI, MGI, EBI and oral hard and soft tissue assessments. Participants had a complete dental prophylaxis before being randomly assigned to one of three treatment groups: (1) LISTERINE® COOL MINT®, an alcohol-containing mouthrinse formulated with four essential oils (ACEO; Kenvue, Summit, NJ, USA [formerly Johnson & Johnson, Skillman, NJ, USA]); (2) an alcohol-free prototype mouthrinse containing four essential oils (AFEO; Kenvue, Summit, NJ, USA); (3) a mouthrinse containing alcohol and no essential oils (negative control; see Supplementary Table S1 for full ingredient lists).

All participants received a manual soft-bristled toothbrush (Concept Curve Winter Series toothbrush), toothpaste (Colgate® Cavity Protection; Colgate-Palmolive, New York, NY, USA) and dental floss (LISTERINE® COOL MINT®; Kenvue, Summit, NJ, USA). At the start of the study, participants were instructed to brush twice daily (morning and evening) in their usual manner using a full ribbon of the supplied toothpaste on the toothbrush provided, and then rinse for 30 s with 20 mL of the assigned mouthrinse for 12 consecutive weeks. No instructions were given for flossing.

Randomisation was based on a block randomisation scheme devised by the trial sponsor. While participants received investigational products in blinded packaging, the investigational mouthrinses were distinguishable in taste and appearance; therefore, the participants were not considered to be fully blinded to the assigned treatment. Personnel dispensing the investigational supplies or supervising their use did not participate in the oral examination of participants. Investigational supplies were kept separate from the site personnel involved in examining the participants; therefore, the dental examiner was blinded to the assigned treatments throughout the study.

### Assessments and outcomes

A trained and calibrated dental examiner performed all examinations in the following order: oral hard and soft tissue examination, MGI, EBI and TPI, consistent with previous trials [11, 12, 26, 27]. This sequence allowed bleeding to be assessed before the red disclosing dye was applied, and careful EBI probing ensured that supragingival plaque was undisturbed.

Gingivitis was scored using the MGI in three gingival sites around all scorable teeth from 0 (absence of inflammation) to 4 (severe inflammation). Bleeding was scored after probing six gingival sites per tooth using the EBI (0 [no bleeding after 30 s]; 1 [bleeding after 30 s] or 2 [immediate bleeding]). Plaque area was scored on six surfaces per tooth using the TPI from 0 (no plaque) to 5 (plaque covering two thirds or more of the surface) (see Supplementary Information for more details).

The primary efficacy endpoints were mean TPI and mean MGI at Week 12. The secondary endpoints were mean TPI at Week 1 and Week 6, mean MGI at Week 6, mean EBI at Week 6 and Week 12, and the percentage of sites that bled (EBI = 1 or 2) at Week 6 and Week 12. See Supplementary Information for additional efficacy analyses.

Participants completed a perception questionnaire at Week 12 to evaluate their experiences of their assigned study rinse and their general oral health awareness (see Supplementary Information).

Safety assessments included oral tissue examinations, conducted at baseline and at subsequent study visits, to monitor oral soft and hard tissue tolerance to the study treatments. Clinically significant findings were recorded as adverse events (AEs), and the examiner assessed the severity and relationship to the investigational product (see Supplementary Information).

### Statistical analysis

The planned sample size of 50 participants per treatment group (150 in total) provides ~80% confidence that the observed proportion of participants experiencing an AE differs from the population proportion by no more than 0.065, provided the population proportion of patients with an AE does not exceed 0.15. This sample size also ensures at least 90% power to detect a between-treatment difference (ACEO versus negative control, or AFEO versus negative control) of 0.44 for MGI and 0.3 for TPI, assuming standard deviations (SDs) of 0.44 and 0.45, respectively, based on previous studies [11], at a two-sided 0.05 significance level.

Demographic and baseline characteristics were compared across treatment groups using analysis of variance (ANOVA) for mean values, and Chi-square test or Fisher’s exact test for categorical values. Efficacy analyses were performed using the full analysis set (i.e. all randomised participants who had both baseline and post-baseline efficacy data). Statistical comparisons for primary and secondary endpoints were based on a mixed model for repeated measures analysis, which included treatment, diabetes type, baseline value, treatment-by-visit interaction and baseline-by-visit interaction. For the perception questionnaire, comparisons were performed using an ANOVA model with investigational product as a factor. The ACEO and AFEO rinses were each compared with the negative control rinse using a two-sided test at a significance level of 0.05. No adjustment for multiple comparisons was applied as the objective was to independently assess the efficacy of ACEO and AFEO rinses relative to the negative control. The safety analysis was based on all participants who signed the consent form for the study.

## Results

**Fig. 1:**
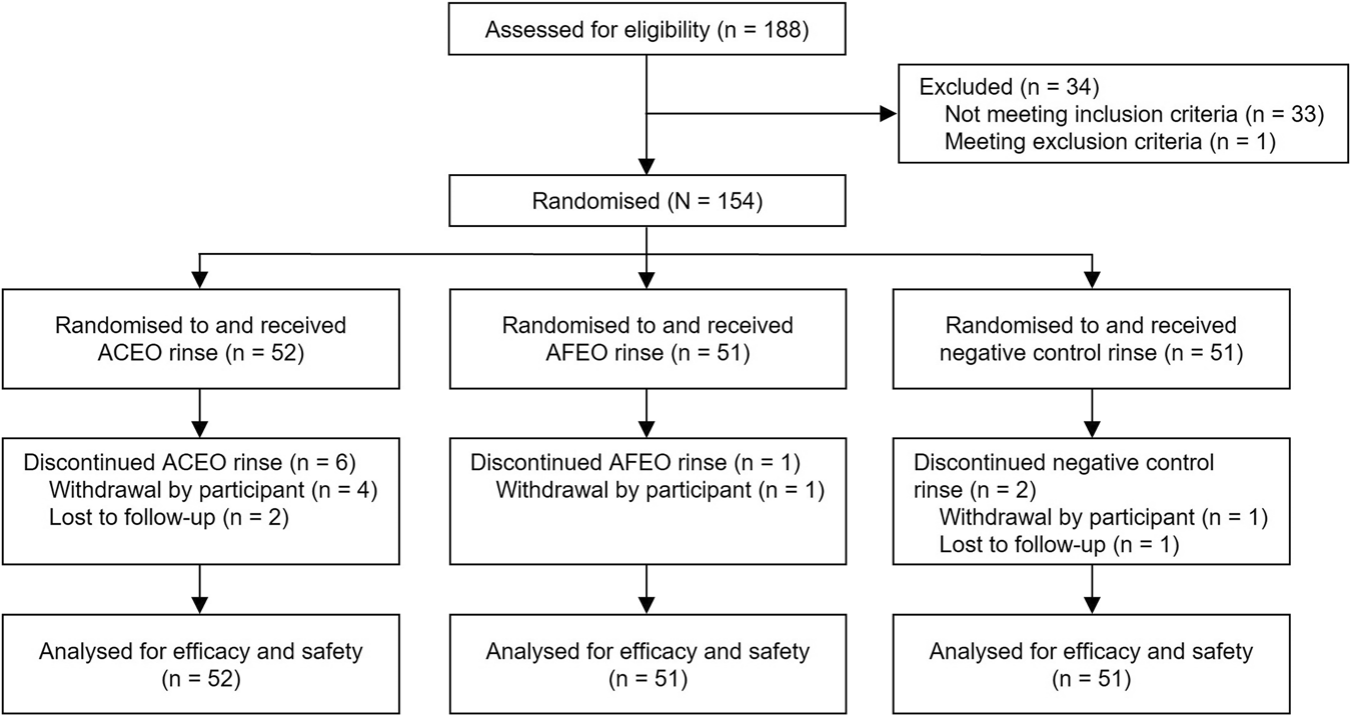
Participant disposition. ACEO alcohol-containing essential oil, AFEO alcohol-free essential oil.

Of the 154 participants who were randomised to receive one of the study mouthrinses, 145 completed the study (Fig. 1).

The overall mean (SD) age of participants was 53.6 (13.3) years, and the majority were female (68.8%), white (79.9%) and non-smokers (97.4%; Table 1). Mean (SD) TPI, MGI and EBI at baseline were 2.95 (0.408), 2.47 (0.309) and 0.31 (0.221), respectively. No significant differences in baseline demographics and disease characteristics were found between the three study groups.

**Table 1:** Baseline demographics and disease characteristics.

	ACEO rinse (*n* = 52)	AFEO rinse (*n* = 51)	Negative control rinse (*n* = 51)	Overall (*N* = 154)	*P* value^a^
**Mean age, years (SD) [range]**	55.7 (12.4) [27–76]	51.3 (15.2) [19–77]	53.9 (11.9) [20–73]	53.6 (13.3) [19–77]	0.255^b^
**Sex**, ***n*** **(%)**					
Male	13 (25.0)	14 (27.5)	21 (41.2)	48 (31.2)	0.163^c^
Female	39 (75.0)	37 (72.5)	30 (58.8)	106 (68.8)	
**Race**, ***n*** **(%)**					
White	39 (75.0)	40 (78.4)	44 (86.3)	123 (79.9)	
Black or African American	12 (23.1)	11 (21.6)	6 (11.8)	29 (18.8)	0.390^d^
Native American	1 (1.9)	0	1 (2.0)	2 (1.3)	
**Smoker**, ***n*** **(%)**					
No	50 (96.2)	49 (96.1)	51 (100)	150 (97.4)	0.547^d^
Yes	2 (3.8)	2 (3.9)	0	4 (2.6)	
**Whole-mouth TPI, mean (SD)**	2.92 (0.407)	2.94 (0.380)	2.99 (0.441)	2.95 (0.408)	0.672^b^
**Whole-mouth MGI, mean (SD)**	2.47 (0.348)	2.47 (0.301)	2.46 (0.280)	2.47 (0.309)	0.981^b^
**Whole-mouth EBI, mean (SD)**	0.32 (0.267)	0.32 (0.228)	0.28 (0.155)	0.31 (0.221)	0.557^b^
**Percentage of sites that bled**^**e**^ **mean (SD)**	23.53 (14.085)	23.73 (13.351)	21.61 (9.618)	22.96 (12.471)	0.640^b^
**Diabetes type**, ***n*** **(%)**					
Type 1	5 (9.6)	6 (11.8)	4 (7.8)	15 (9.7)	0.844^d^
Type 2	47 (90.4)	45 (88.2)	47 (92.2)	139 (90.3)	

*ACEO* alcohol-containing essential oil, *AFEO* alcohol-free essential oil, *ANOVA* analysis of variance, *EBI* Expanded Bleeding Index, *MGI* Modified Gingival Index, *SD* standard deviation, *TPI* Turesky modification of the Quigley–Hein Plaque Index.

^a^Across all three treatment groups.

^b^Based on an ANOVA model with a term for treatment.

^c^Based on Chi-square test.

^d^Based on Fisher’s exact test.

^e^EBI = 1 or 2.

### Efficacy

#### Primary endpoints

**Fig. 2:**
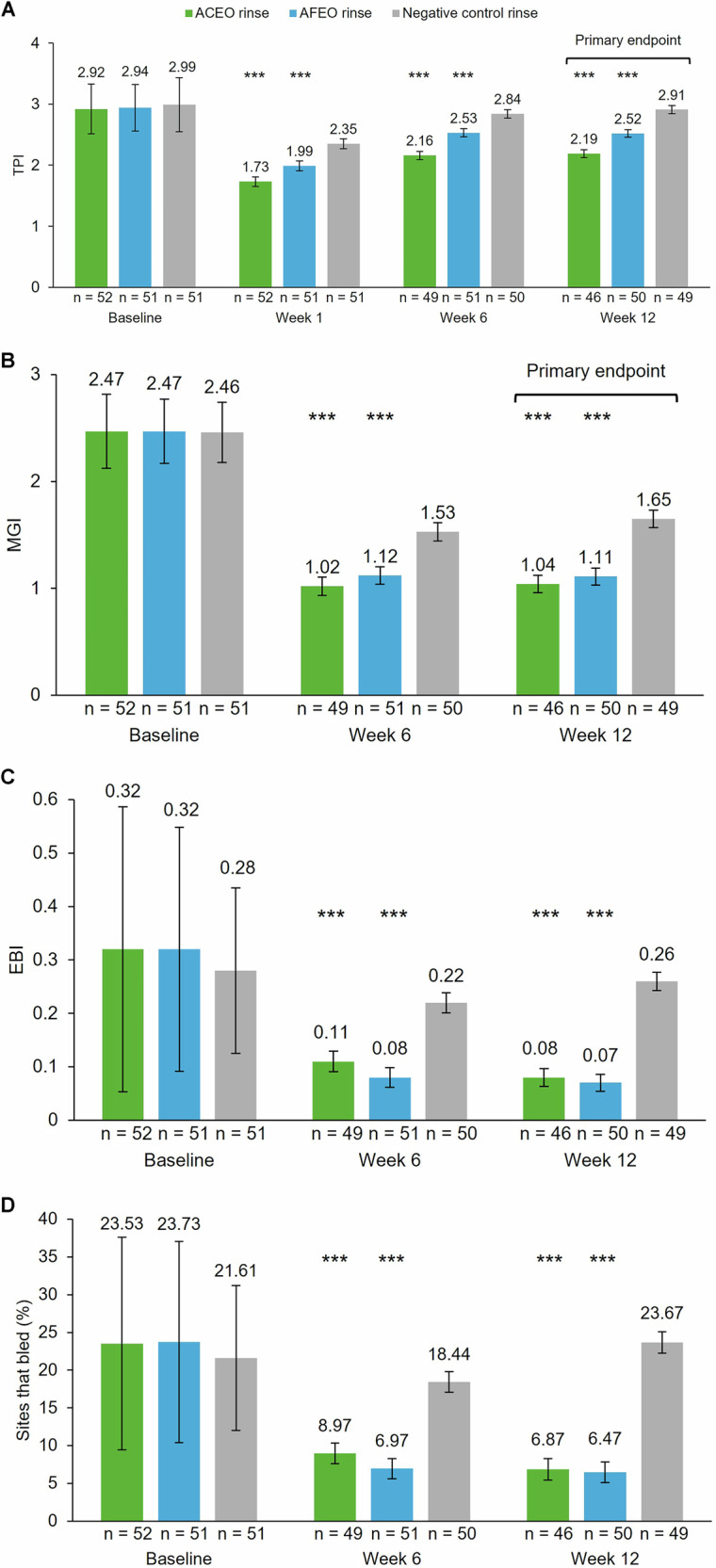
Effects of essential‑oil‑containing mouthrinses on plaque, gingivitis and gingival bleeding over time. Mean **A** TPI, **B** MGI, **C** EBI and **D** percent of sites that bled (EBI = 1 or 2). ***p < 0.001 versus the negative control rinse. Baseline values are arithmetic means (SD); post-baseline values are adjusted least‑squares means (SE). ACEO alcohol-containing essential oil, AFEO alcohol-free essential oil, EBI Expanded Bleeding Index, MGI Modified Gingival Index, SD standard deviation, SE standard error, TPI Turesky modification of the Quigley–Hein Plaque Index.

At Week 12, the ACEO and AFEO groups both demonstrated significantly lower TPI compared with the negative control group (mean [standard error (SE)]: 2.19 [0.064] and 2.52 [0.062] versus 2.91 [0.065]; *p* < 0.001 for both comparisons; Fig. 2A), representing mean differences of −24.7% and −13.2% for ACEO and AFEO, respectively, relative to the control group (Supplementary Table S2).

Similarly, MGI at Week 12 was significantly lower in the ACEO and AFEO groups compared with the negative control group (mean [SE]: 1.04 [0.082] and 1.11 [0.079] versus 1.65 [0.082]; *p* < 0.001 for both comparisons; Fig. 2B), equivalent to mean differences of −37.2% and −32.8%, respectively (Supplementary Table S3).

#### Secondary endpoints

Both the ACEO and AFEO groups had significantly lower mean TPI and MGI scores by Week 1 and Week 6, respectively, than the negative control group (Fig. 2A and B, Supplementary Tables S2 and S3).

Similarly, the mean EBI score and percentage of sites that bled were significantly lower in the ACEO and AFEO groups versus the negative control group at Week 6 and Week 12 (Fig. 2C and D, Supplementary Tables S4 and S5).

#### Additional efficacy analyses

To evaluate outcomes indicative of clinically healthy oral status, analyses were conducted on the percentage of sites with no or minimal plaque, gingivitis and/or bleeding. The percentage of sites with no or minimal plaque (TPI = 0 or 1) at Week 12 was significantly higher in the ACEO and AFEO groups than the negative control group (mean [SE]: 18.29% [1.848] and 10.41% [1.786] versus 3.17% [1.858], respectively; *p* < 0.001 for both comparisons).

The percentage of sites with no or minimal gingivitis (MGI = 0 or 1) at Week 12 was also significantly higher in the ACEO and AFEO groups than the negative control group (mean [SE]: 66.31% [4.524] and 61.20% [4.402] versus 40.40% [4.540], respectively; *p* < 0.001 for both comparisons).

Furthermore, the percentage of sites that had both no/minimal gingivitis (MGI = 0 or 1) and no gingival bleeding (EBI = 0) at Week 12 was significantly higher in the ACEO and AFEO groups than the negative control group (mean [SE]: 58.32% [4.540] and 53.74% [4.422] versus 31.19% [4.555], respectively; *p* < 0.001 for both comparisons).

#### Participant survey

**Fig. 3:**
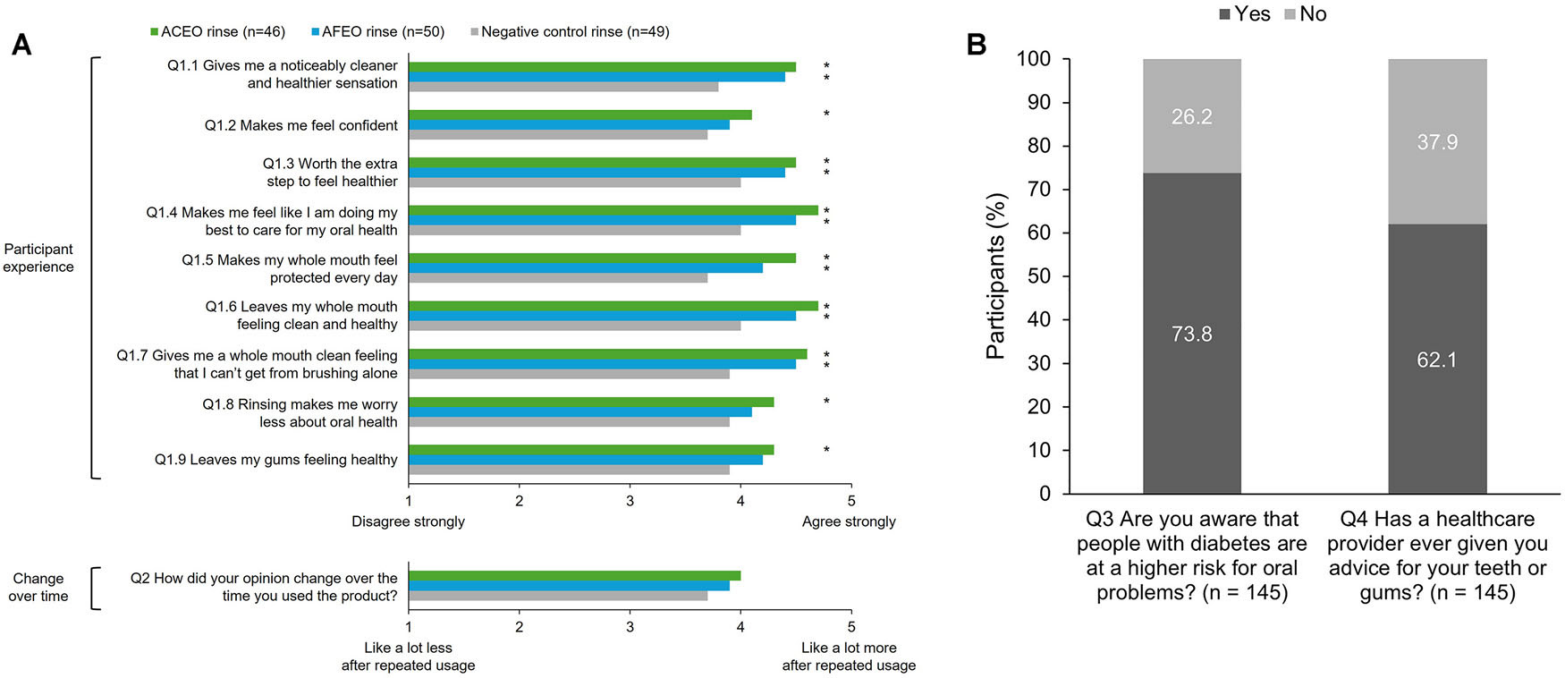
Participants' responses to survey questions. **A** Experiences with the assigned study mouthrinses; **B** oral health awareness. *p < 0.05 versus the negative control rinse. ACEO alcohol-containing essential oil, AFEO alcohol-free essential oil, Q question.

ACEO and AFEO users reported significantly better experiences with the essential-oil-containing rinses, compared with the negative control rinse, across multiple domains; the ACEO group showed significantly higher agreement across all nine statements, while the AFEO group demonstrated this in six statements (Fig. 3A). A numerically higher proportion of participants reported increased preference for ACEO and AFEO rinses over time, compared with the negative control, although this did not reach statistical significance.

Overall, 38/145 participants (26.2%) were unaware that they had an elevated risk of oral problems because of their diabetes compared with the general population (Fig. 3B). In addition, 55/145 participants (37.9%) reported receiving no advice on their teeth and gums from their healthcare provider prior to the study.

### Safety

Overall, six (3.9%) participants reported at least one treatment-emergent AE, all of which were mild or moderate in intensity and resolved without treatment (Table 2). No deaths, serious AEs or AEs leading to study discontinuation were reported.

**Table 2:** Treatment-emergent AEs.

*n* (%)	ACEO rinse (*n* = 52)	AFEO rinse (*n* = 51)	Negative control rinse (*n* = 51)
**Participants with** ≥ **1 AE**	**1 (1.9)**	**3 (5.9)**	**2 (3.9)**
**AEs of mild intensity**	**1 (1.9)**	**2 (3.9)**	**1 (2.0)**
Dry mouth	–	1 (2.0)	–
Oral mucosal exfoliation	1 (1.9)	–	–
Tongue coated	–	1 (2.0)	–
Lip dry	–	–	1 (2.0)
**AEs of moderate intensity**	**1 (1.9)**	**1 (2.0)**	**2 (3.9)**
Dry mouth	–	–	1 (2.0)
Oral mucosal exfoliation	–	–	1 (2.0)
Tongue coated	1 (1.9)	–	–
Tongue erythema	–	1 (2.0)	–
**AEs of severe intensity**	–	–	**2 (3.9)**
**Participants with study-product-related AEs**	**1 (1.9)**	**2 (3.9)**	**2 (3.9)**
Dry mouth	–	1 (2.0)	1 (2.0)
Oral mucosal exfoliation	1 (1.9)	–	1 (2.0)
Tongue erythema	–	1 (2.0)	–

AE classification: mild—symptoms that are easily tolerated, causing minimal discomfort and not interfering with usual function or everyday activities; moderate—sufficient discomfort is present to cause interference to some extent with usual function or everyday activity; severe—extreme distress, causing significant impairment of functioning or incapacitation, interferes significantly with usual function, prevents everyday activities.

*AE* adverse event, *ACEO* alcohol-containing essential oil, *AFEO* alcohol-free essential oil.

Five participants (3.2%) experienced AEs that were judged to be related to the study product: dry mouth (n = 1 each for AFEO and negative control rinses); oral mucosal exfoliation (*n* = 1 each for ACEO and negative control rinses); and tongue erythema (*n* = 1 for AFEO rinse).

## Discussion

This was the first clinical trial, to our knowledge, to evaluate the efficacy and safety of essential-oil-containing mouthrinses in people with diabetes. We found that ACEO and AFEO rinses significantly reduced plaque and gingivitis/gingival bleeding within 1 and 6 weeks, respectively, compared with a negative control rinse, and these benefits were maintained at 12 weeks. At 12 weeks, the ACEO and AFEO groups showed substantial reductions in plaque, gingivitis and gingival bleeding scores, as well as the percentage of gingival sites that bled, compared with the negative control. The percentage of sites with no or minimal plaque was nearly six-fold higher with ACEO and over three-fold higher with AFEO compared with the negative control. Notably, the percentage of sites that had both no/minimal gingivitis and no gingival bleeding – a highly stringent measure of whole-mouth gingival health – was significantly higher with ACEO and AFEO than with negative control (*p* < 0.001 for both).

Our findings align with previous results in people without diabetes where essential-oil-containing mouthrinses, used in conjunction with mechanical cleaning methods, significantly reduced plaque, gingivitis and gingival bleeding over a 12-week period compared with mechanical methods alone [26]. These findings are important because people with diabetes face a higher risk of periodontal disease but are also less likely to seek preventive dental care than those without diabetes, in part because of financial constraints [1]. Taken together, the demonstrated efficacy, accessibility and low cost of essential-oil-containing mouthrinses support their use as a valuable adjunctive therapy for oral hygiene in people with diabetes. Both alcohol-containing and alcohol-free formulations demonstrated efficacy in people with diabetes, mirroring findings in the general population [12] and providing clinicians with flexibility in recommending alcohol-free products for those with religious or other concerns about alcohol.

Participants reported significantly better experiences with the essential-oil-containing rinses than with the negative control rinse, noting enhanced sensations of cleanliness, oral health and protection and a whole-mouth clean feeling beyond brushing alone. The ACEO rinse also increased feelings of confidence and of healthy gums, and reduced worries about oral health. Such positive user experiences could potentially enhance adherence to oral care routines and facilitate lasting habit changes [20, 21], impacting real-world effectiveness.

Our survey also revealed a knowledge gap among people with diabetes regarding their elevated oral health risks and a communication deficit with healthcare providers. While the American Diabetes Association provides basic oral care recommendations for people with diabetes [28], our survey suggests that many patients do not receive adequate advice. Given the bidirectional relationship between diabetes mellitus and periodontal disease [2], a multidisciplinary approach is crucial. The entire patient care team, including nurses, diabetes practitioners and dental professionals, should actively enquire about oral health status in patients with diabetes and implement guideline recommendations for comprehensive oral health education and regular periodontal examinations [3, 29]. Early intervention is crucial since gingivitis is reversible and timely treatment can prevent advancement to irreversible periodontal disease.

The essential-oil-containing mouthrinses presented good oral soft tissue tolerance after 12 weeks of use in people with diabetes, with a safety profile similar to that observed in the general population [11, 12, 26], helping to address a paucity of data in this population. No AEs related to oral tissue discoloration or taste alterations were reported during the study, although it should be noted that these outcomes were not specifically evaluated as part of our protocol.

Periodontal disease can affect glycaemic control through various mechanisms, including the spread of periodontal bacteria and bacterial byproducts into systemic circulation and the release of inflammatory cytokines from the periodontium [30, 31], leading to the potential initiation or worsening of insulin resistance [2]. Previous studies have shown that effective periodontal disease management leads to clinically meaningful, statistically significant improvements in glycaemic control in people with type 2 diabetes (data in type 1 diabetes are lacking) [3, 31]. Furthermore, effective treatment of gingivitis with an essential-oil-containing mouthrinse has been shown to improve oral health-related quality of life in people with diabetes, compared with a placebo rinse, suggesting potential broader benefits of effective oral health interventions in this population [32]. As glycaemic or systemic outcomes were not evaluated in the current study, confirmation of any such benefits associated with use of essential-oil-containing mouthrinses in this population requires further study.

While local and international clinical guidelines on mouthrinse use in people with diabetes are generally lacking, German guidelines advocate for a cross-disciplinary approach, recommending antibacterial mouthrinses, as a supplement to mechanical cleaning, to reduce dental biofilm and thus prevent gingivitis in patients across all areas of dental and medical care [33]. Future international guidelines should consider a similar broad perspective to facilitate early interventions and improve outcomes across therapy areas.

Our trial had several limitations. The scope did not include statistical comparisons between the two essential-oil-containing rinses, precluding the identification of potential superiority between formulations. The use of a negative‑control rinse rather than an active comparator, such as CPC or CHX, also prevents comparison between the performance of essential‑oil‑containing mouthrinses and other chemotherapeutic agents. Additionally, subgroup analyses were conducted to evaluate efficacy endpoints by diabetes type and HbA1c levels; however, small sample sizes in certain subgroups (particularly type 1 diabetes) limited the interpretation of results, hence they are not presented here. The 12-week duration of the study, while consistent with previous research [11, 26] and American Dental Association guidelines [27], may not fully capture long-term effects or sustainability of the observed outcomes. Participant blinding was incomplete because the rinses differed in taste and appearance, raising the possibility of expectation bias in subjective outcomes such as the perception questionnaire. The oral health awareness survey also warrants caution as it was exploratory, not validated, and therefore may have limited generalisability. Lastly, we did not investigate the potential impact of improved periodontal health on glycaemic control or diabetes-related complications. Future studies with larger sample sizes, a larger number of people with type I diabetes, active comparators, longer follow-up and assessments of systemic health outcomes are needed to provide a more comprehensive understanding of the benefits of essential-oil-containing mouthrinses in people with diabetes. Future work could also assess brushing and flossing behaviours to better contextualise their potential influence on periodontal outcomes.

## Conclusions

Overall, the results of our study indicate that essential-oil-containing mouthrinses may provide adjunctive benefits, compared with a negative control rinse, in people with diabetes and mild-to-moderate gingivitis and plaque, when used alongside mechanical oral hygiene. This approach is consistent with clinical guidelines for the management of periodontal disease, which state that antiseptic agents may be offered to individuals as part of a personalised treatment approach [34]. Our participant survey demonstrated that there is an unmet need for patient education on the elevated risks of oral complications in diabetes and on the need for regular preventative oral care. For people with diabetes, integrating essential-oil-containing mouthrinses into their oral care routine, alongside multidisciplinary support and patient education, may potentially contribute to improved overall health outcomes, with further study warranted to fully explore these effects.

## Supplementary information

Supplement to: Efficacy and safety of essential-oil-containing mouthrinses for plaque and gingivitis control in people with diabetes: an examiner blind, randomised controlled trial

## Data Availability

Johnson & Johnson Consumer Inc. has an agreement with the Yale Open Data Access (YODA) Project to serve as the independent review panel for evaluation of requests for clinical study reports and participant level data from investigators and physicians for scientific research that will advance medical knowledge and public health. Requests for access to the study data can be submitted through the YODA Project site at http://yoda.yale.edu.
