# Supplementary material for: Efficacy and safety of essential-oil-containing mouthrinses for plaque and gingivitis control in people with diabetes: an examiner-blind, randomised controlled trial
Source: BDJ Open. 2026 Jun 22;12:63. doi: 10.1038/s41405-026-00420-5 (PMC13287467; doi:10.1038/s41405-026-00420-5)
Supplement: Supplementary file 1 — Supplement to: Efficacy and safety of essential-oil-containing mouthrinses for plaque and gingivitis control in people with diabetes: an examiner blind, randomised controlled trial [file 41405_2026_420_MOESM1_ESM.pdf]

Supplement to: Efficacy and safety of essential-oil-containing mouthrinses for plaque and gingivitis control in people with diabetes: an examiner-blind, randomised controlled trial

Table of contents

1 Protocol amendment 3

2 Eligibility criteria 3

3 MGI, EBI and TPI scoring 6

4 Additional efficacy analyses 6

5 Perception questionnaire 7

6 Adverse event classification 9

7 Supplementary tables 10

1 Protocol amendment

In accordance with the approved protocol (25 November 2019), participants originally self-reported whether they had been diagnosed with type 1 diabetes or type 2 diabetes. However, after trial completion, it was noted that substantially more participants self-reported type 1 diabetes than type 2 diabetes, which is inconsistent with prevalences in the general population. The protocol was amended (16 December 2019) and a follow-up assessment was conducted in April 2022, in which study participants were interviewed by the study site physician, who also examined their diabetes medications, to verify the diagnosis of type 1 diabetes or type 2 diabetes. The verification process showed that many participants had inaccurately self-reported their diabetes type; therefore, the clinical trial results were re-analysed when necessary, and those results are presented here. It should also be noted that one participant died before the updated diabetes type information was acquired; their originally reported diabetes type was used in the data analysis.

2 Eligibility criteria

- Inclusion criteria
  - Adults, ≥18 years of age, in good general and oral health
  - Adequate oral hygiene (ie brush teeth daily and exhibit no signs of oral neglect)
  - Able to comprehend and follow the requirements and restrictions of the study (including willingness to use the assigned study products per instructions, availability on scheduled visit dates, and likeliness of completing the clinical study) based upon research site personnels' assessment
  - Able to read and understand study documents
  - Evidence of a personally signed and dated informed consent document indicating the participant (or legally acceptable representative) has been informed of all pertinent aspects of the trial
  - Negative pregnancy urine tests (females of childbearing potential only)
  - Females of childbearing potential must be using a medically acceptable method of birth control for at least 1 month prior to Visit 1 and agree to continue using this method during their participation in the study

- A minimum of 20 gradable teeth, including four molars, with scorable facial and lingual surfaces. Teeth that are grossly carious, extensively restored, orthodontically banded, abutments, exhibiting severe generalised cervical and/or enamel abrasion, teeth with veneers, or third molars will not be included in the tooth count
- Type 1 or type 2 diabetes
- A glycated haemoglobin (HbA1c) level <7.0% for type 1 diabetes or <8.0% for type 2 diabetes
- Mean modified gingival index ≥1.85 at baseline
- Turesky modification of the Quigley–Hein plaque index ≥1.95 per the six-site index at baseline
- Absence of significant oral soft tissue pathology, excluding plaque-induced gingivitis, based on a visual examination and at the discretion of the investigator
- Absence of moderate/advanced periodontitis based on a clinical examination and at the discretion of the dental examiner
- Absence of fixed or removable orthodontic appliance, removable partial dentures, bruxism, or temporomandibular joint device.

• Exclusion criteria
  - History of significant adverse effects, including sensitivities or suspected allergies, following use of oral hygiene products such as toothpastes, mouthrinses and red food dye
  - Known allergy or sensitivity or history of significant adverse effects to any of the investigational product and/or product ingredients (or other ingredients in the products)
  - Dental prophylaxis within 4 weeks prior to the baseline visit
  - More than three sites having periodontal pocket depths of 5 mm or any sites that are greater than 5 mm in depth
  - History of medical conditions requiring prophylactic antibiotic coverage prior to invasive dental procedures
  - A recent history of hypoglycaemia requiring medical intervention within the past 30 days
  - Use of antibiotics, anti-inflammatory or anticoagulant therapy, phenytoin sodium or diphenylhydantoin, calcium channel blockers, cyclosporin A, or

immunostimulants/immunomodulators during the study or within 1 month prior to the baseline exam. Intermittent use of certain anti-inflammatory medication is acceptable at the discretion of the investigator

– Use of chemotherapeutic antiplaque/antigingivitis products, such as triclosan, essential oils, cetylpyridinium chloride, stannous fluoride, zinc or chlorhexidine-containing mouth rinses and/or toothpastes, within 4 weeks prior to the screening/baseline exam
– Self-reported pregnancy or lactation (this criterion is because oral tissue changes related to pregnancy and nursing can affect interpretation of study results)
– Self-reported smokeless tobacco user (eg snuff, chewing tobacco)
– Self-reported vaping and e-cigarette usage
– Suspected alcohol or substance abuse (eg amphetamines, benzodiazepines, cocaine, marijuana, opiates)
– Any significant unstable or uncontrolled medical condition that may interfere with participation in the study, at the discretion of the investigator
– Participation in any clinical trial within 30 days of the baseline exam
– Diagnosed temporomandibular joint dysfunction
– Wearing of intraoral devices during the day or evening, such as orthodontic retaining appliances, orthodontic tooth movement appliances, partial dentures, splints, or apnea appliances
– Participants who were previously screened and ineligible for the current study, or who were randomised to receive investigational product
– People related to those persons involved directly or indirectly with the conduct of this study (ie principal investigator, subinvestigators, study coordinators, other site personnel)
– Other severe acute or chronic medical or psychiatric condition or laboratory abnormality that may increase the risk associated with study participation or investigational product administration, or may interfere with the interpretation of study results and, in the judgment of the investigator, would make the individual inappropriate for entry into this study.

3 MGI, EBI and TPI scoring

Gingivitis was scored using the MGI on the buccal and lingual marginal gingivae and interdental papillae of all scorable teeth as follows: 0 (absence of inflammation); 1 (mild inflammation of any portion of the gingival unit); 2 (mild inflammation of the entire gingival unit); 3 (moderate inflammation of the gingival unit); or 4 (severe inflammation of the gingival unit).

To assess gingival bleeding, a periodontal probe with a 0.5 mm diameter tip was inserted into the gingival crevice and swept from distal to mesial around the tooth at an angle of approximately 60°, while in contact with the sulcular epithelium. Bleeding was scored after probing six gingival areas (distobuccal, midbuccal, mesiobuccal, distolingual, midlingual, and mesiolingual) around each tooth using the EBI as follows: 0 (no bleeding after 30 seconds); 1 (bleeding after 30 seconds); or 2 (immediate bleeding).

Plaque area was scored using the TPI on six surfaces (distobuccal, midbuccal, mesiobuccal, distolingual, midlingual, and mesiolingual) of all scorable teeth as follows: 0 (no plaque); 1 (separate flecks or discontinuous band of plaque at the gingival margin); 2 (up to 1 mm continuous band of plaque at the gingival margin); 3 (band of plaque wider than 1 mm but less than 1/3 of surface); 4 (plaque covering 1/3 or more, but less than 2/3 of surface); or 5 (plaque covering 2/3 or more of surface). For each patient, individual site scores were recorded using the three clinical indices (MGI, TPI and EBI), and an average score was calculated for each index.

4 Additional efficacy analyses

Additional efficacy analyses evaluated the percentages of sites with no or minimal plaque (TPI = 0 or 1), no or minimal gingivitis (MGI = 0 or 1), and sites exhibiting both no/minimal gingivitis and no gingival bleeding (EBI = 0) at Week 12. For this last analysis of combined MGI and EBI measures, the single MGI score on the lingual interdental area was paired with the EBI scores from the mesiolingual/distolingual regions of adjacent teeth, with an equivalent pairing approach applied for the buccal surface.

5 Perception questionnaire

PARTICIPANT INSTRUCTIONS:

PLEASE READ PRIOR TO COMPLETING THE QUESTIONNAIRE

Thank you for agreeing to participate in this survey. Your input is very valuable and helps in the development of better products. Please remember you are giving your opinions, so there is no right or wrong answer.

You will be asked a few questions about the mouthrinse product you were given after using it for 12 weeks. Some of the questions included will ask you to consider how the mouth rinse made you feel, how much you liked or disliked the mouth rinse overall, as well as some other general questions.

When completing the questionnaire, please circle the number which corresponds to the phrase which best answers each question. We are looking for your own personal opinions about the product.

Q1. Thinking about the mouthwash you used in this study; how much do you agree or disagree with the following statements. For each statement, please use the scale below to tell us how much you agree or disagree that each statement describes the mouthrinse product **(Select one response per question)**

Question #	Question	Agree strongly	Agree somewhat	Neither agree nor disagree	Disagree somewhat	Disagree strongly
1	Gives me a noticeably cleaner and healthier sensation	5	4	3	2	1
2	Makes me feel confident	5	4	3	2	1
3	Worth the extra step to feel healthier	5	4	3	2	1
4	Makes me feel like I am doing my best to care for my oral health	5	4	3	2	1
5	Makes my whole mouth feel protected everyday	5	4	3	2	1
6	Leaves my whole mouth feeling clean and healthy	5	4	3	2	1
7	Gives me a whole mouth clean feeling that I can't get from brushing alone	5	4	3	2	1
8	Rinsing makes me worry less about my oral health	5	4	3	2	1
9	Leaves my gums feeling healthy	5	4	3	2	1

Q2. How did your opinion change, if at all, over the time you used this mouthwash product? Did you…? **(Please circle one)**

5	Like it much more after repeated usage
4	Like it a little more after repeated usage
3	Like it about the same throughout
2	Like it a little less after repeated usage
1	Like it a lot less after repeated usage

Q3. Are you aware that people with diabetes are at a higher risk for oral health problems? **(Please check one)**	Yes ☐<br>No ☐
Q4. Has a healthcare provider (dentist, hygienist, physician) ever given you any specific advice/instructions about caring for your teeth or gums? **(Please check one)**	Yes ☐<br>No ☐

6 Adverse event classification

Adverse events were classed as mild (symptoms that are easily tolerated, causing minimal discomfort and not interfering with usual function or everyday activities), moderate (sufficient discomfort is present to cause interference to some extent with usual function or everyday activity) or severe (extreme distress, causing significant impairment of functioning or incapacitation; interferes significantly with usual function; prevents everyday activities).

7 Supplementary tables

Table S1 Mouthrinse ingredients

ACEO rinse	AFEO rinse	Negative control rinse
Water	Water	Water
Eucalyptol	Eucalyptol	Colour
Menthol	Menthol	Flavour
Methyl salicylate	Methyl salicylate	Ethanol
Thymol	Thymol	
Flavour	Flavors	
Colour	Colour	
Poloxamer	Poloxamer	
Benzoic acid	Benzoic acid	
Sorbitol	Sorbitol	
Sodium benzoate	Sodium benzoate	
Sodium saccharin	Sodium saccharin	
Ethanol	Propylene glycol	
	Betain	
	Sucralose	

ACEO=alcohol-containing essential oil; AFEO=alcohol-free essential oil.

Table S2 TPI at baseline, Week 1, Week 6 and Week 12

Week	Arm	n/N†	TPI‡	Comparison with negative control rinse		
				**Difference** (SE) [95% CI]	**Percentage difference**	***p* value§**
Baseline	ACEO rinse	52/52	2.92 (0.407)	–	–	–
	AFEO rinse	51/51	2.94 (0.380)	–	–	–
	Negative control rinse	51/51	2.99 (0.441)	–	–	–
1	ACEO rinse	52/52	1.73 (0.080)	−0.62 (0.101) [−0.819, −0.420]	−26.3	<0.001
	AFEO rinse	51/51	1.99 (0.080)	−0.36 (0.101) [−0.563, −0.162]	−15.4	<0.001
	Negative control rinse	51/51	2.35 (0.082)	–	–	–
6	ACEO rinse	49/52	2.16 (0.067)	−0.68 (0.079) [−0.838, −0.525]	−24.0	<0.001
	AFEO rinse	51/51	2.53 (0.066)	−0.31 (0.079) [−0.468, −0.156]	−11.0	<0.001
	Negative control rinse	50/51	2.84 (0.068)	–	–	–
12	ACEO rinse	46/52	2.19 (0.064)	−0.72 (0.074) [−0.865, −0.573]	−24.7	<0.001
	AFEO rinse	50/51	2.52 (0.062)	−0.38 (0.073) [−0.528, −0.240]	−13.2	<0.001
	Negative control rinse	49/51	2.91 (0.065)	–	–	–

†n = number of participants with data at the reported timepoint. N = number of randomised participants; ‡Baseline values are arithmetic means (SD); post-baseline values are adjusted least squares means (SE); §*p* values are based on a mixed model for repeated analysis that included treatment, diabetes type, baseline value, treatment-by-visit interaction, and baseline-by-visit interaction. ACEO=alcohol-containing essential oil; AFEO=alcohol-free essential oil; CI=confidence interval; SD=standard deviation; SE=standard error; TPI=Turesky modification of the Quigley–Hein Plaque Index.

Table S3 MGI at baseline, Week 6 and Week 12

Week	Arm	n/N†	MGI‡	Comparison with the negative control rinse		
				**Difference** (SE) [95% CI]	**Percentage difference**	***p* value§**
Baseline	ACEO rinse	52/52	2.47 (0.348)	–	–	–
	AFEO rinse	51/51	2.47 (0.301)	–	–	–
	Negative control rinse	51/51	2.46 (0.280)	–	–	–
6	ACEO rinse	49/52	1.02 (0.084)	−0.51 (0.098) [−0.705, −0.316]	−33.5	<0.001
	AFEO rinse	51/51	1.12 (0.082)	−0.41 (0.097) [−0.601, −0.216]	−26.8	<0.001
	Negative control rinse	50/51	1.53 (0.085)	–	–	–
12	ACEO rinse	46/52	1.04 (0.082)	−0.62 (0.094) [−0.801, −0.429]	−37.2	<0.001
	AFEO rinse	50/51	1.11 (0.079)	−0.54 (0.093) [−0.726, −0.360]	−32.8	<0.001
	Negative control rinse	49/51	1.65 (0.082)	–	–	–

†n = number of participants with data at the reported timepoint. N = number of randomised participants; ‡Baseline values are arithmetic means (SD); post-baseline values are adjusted least squares means (SE); §*p* values are based on a mixed model for repeated analysis that included treatment, diabetes type, baseline value, treatment-by-visit interaction, and baseline-by-visit interaction. ACEO=alcohol-containing essential oil; AFEO=alcohol-free essential oil; CI=confidence interval; MGI=Modified Gingival Index; SD=standard deviation; SE=standard error.

Table S4 EBI at baseline, Week 6 and Week 12

Week	Arm	n/N†	EBI‡	Comparison with the negative control rinse		
				**Difference** (SE) [95% CI]	**Percentage difference**	***p* value**[c]
Baseline	ACEO rinse	52/52	0.32 (0.267)	–	–	–
	AFEO rinse	51/51	0.32 (0.228)	–	–	–
	Negative control rinse	51/51	0.28 (0.155)	–	–	–
6	ACEO rinse	49/52	0.11 (0.019)	−0.12 (0.023) [−0.161, −0.071]	−51.6	<0.001
	AFEO rinse	51/51	0.08 (0.018)	−0.14 (0.023) [−0.189, −0.100]	−64.4	<0.001
	Negative control rinse	50/51	0.22 (0.019)	–	–	–
12	ACEO rinse	46/52	0.08 (0.017)	−0.18 (0.019) [−0.214, −0.138]	−68.7	<0.001
	AFEO rinse	50/51	0.07 (0.016)	−0.19 (0.019) [−0.223, −0.149]	−72.6	<0.001
	Negative control rinse	49/51	0.26 (0.017)	–	–	–

†n = number of participants with data at the reported timepoint. N = number of randomised participants; ‡Baseline values are arithmetic means (SD); post-baseline values are adjusted least squares means (SE); §*p* values are based on a mixed model for repeated analysis that included treatment, diabetes type, baseline value, treatment-by-visit interaction, and baseline-by-visit interaction. ACEO=alcohol-containing essential oil; AFEO=alcohol-free essential oil; CI=confidence interval; EBI=expanded bleeding index; SD=standard deviation; SE=standard error.

Table S5 Percentage of sites that bled (EBI = 1 or 2) at baseline, Week 6 and Week 12

				Comparison with the negative control rinse		
**Week**	**Arm**	**n/N†**	**Percentage of sites that bled‡**	**Difference** (SE) [95% CI]	**Percentage difference**	***p* value§**
Baseline	ACEO rinse	52/52	23.53 (14.085)	–	–	–
	AFEO rinse	51/51	23.73 (13.351)	–	–	–
	Negative control rinse	51/51	21.61 (9.618)	–	–	–
6	ACEO rinse	49/52	8.97 (1.368)	−9.46 (1.598) [−12.621, −6.305]	−51.3	<0.001
	AFEO rinse	51/51	6.97 (1.332)	−11.46 (1.588) [−14.601, −8.325]	−62.2	<0.001
	Negative control rinse	50/51	18.44 (1.384)	–	–	–
12	ACEO rinse	46/52	6.87 (1.430)	−16.80 (1.694) [−20.150, −13.452]	−71.0	<0.001
	AFEO rinse	50/51	6.47 (1.379)	−17.21 (1.669) [−20.507, −13.909]	−72.7	<0.001
	Negative control rinse	49/51	23.67 (1.430)	–	–	–

†n = number of participants with data at the reported timepoint. N = number of randomised participants; ‡Baseline values are arithmetic means (SD); post-baseline values are adjusted least squares means (SE); §*p* values are based on a mixed model for repeated analysis that included treatment, diabetes type, baseline value, treatment-by-visit interaction, and baseline-by-visit interaction. ACEO=alcohol-containing essential oil; AFEO=alcohol-free essential oil; CI=confidence interval; EBI=expanded bleeding index; SD=standard deviation; SE=standard error.
